# Picopore: A tool for reducing the storage size of Oxford Nanopore Technologies datasets without loss of functionality

**DOI:** 10.12688/f1000research.11022.3

**Published:** 2017-09-28

**Authors:** Scott Gigante

**Affiliations:** 1 Walter & Eliza Hall Institute of Medical Research, Parkville, Victoria, 3121, Australia

**Keywords:** DNA Sequencing, Genome Informatics, Nanopore Sequencing, Compression, Data Storage

## Abstract

Oxford Nanopore Technologies' (ONT's) MinION and PromethION long-read sequencing technologies are emerging as genuine alternatives to established Next-Generation Sequencing technologies. A combination of the highly redundant file format and a rapid increase in data generation have created a significant problem both for immediate data storage on MinION-capable laptops, and for long-term storage on lab data servers. We developed Picopore, a software suite offering three methods of compression. Picopore's lossless and deep lossless methods provide a 25% and 44% average reduction in size, respectively, without removing any data from the files. Picopore's raw method provides an 88% average reduction in size, while retaining biologically relevant data for the end-user. All methods have the capacity to run in real-time in parallel to a sequencing run, reducing demand for both immediate and long-term storage space.

## Introduction

Oxford Nanopore Technologies’ (ONT’s) nanopore sequencing technology MinION provides a high-throughput, low-cost alternative to traditional Next-Generation Sequencing (NGS) technologies ^1^. The sequencing device itself is handheld and connects by USB to a laptop computer. Together with all equipment and reagents required for DNA library preparation, the equipment required to use MinION is minimal; entire laboratories have even been transported overseas in a suitcase, allowing a versatile and agile approach towards DNA and RNA sequencing ^2^.

Over the course of ONT’s Early Access Program, several improvements in software and chemistry have led to a rapid increase in yield, through an increase in average read length, an improvement in basecalling accuracy rates and an increase in total number of reads. In October 2015, the MinION Analysis and Reference Consortium (MARC), using R7.3 flow cells and SQK-MAP005 (2D) chemistry, reported a median of 60,600 reads with a median yield of 650,000 events across 20 MinION experiments ^3^. In contrast, ONT claim to have obtained a total base yield of 17 gigabases using an R9.4 flowcell on the latest version of their MinKNOW software ( https://nanoporetech.com/about-us/news/minion-software-minknow-upgraded-enable-increased-data-yield-other-benefits). Dramatic increases in MinION flowcell throughput have highlighted the need for reduced per-base data handling and storage demands now and into the future.

The concerns over data storage extend beyond the data generation capabilities of a single flowcell. Recent attempts to perform *de novo* assembly of eukaryotic genomes have combined the data generated by multiple flowcells in order to gain sufficient coverage of the genome ^4^. To this end, ONT have begun the precommercial release of the PromethION, a benchtop nanopore sequencing device with 48 flowcells. In addition, each of these flowcells contains 3000 channels, as opposed to the 512 channels in a single MinION flowcell. Data generation from one PromethION unit is projected at up to 6 terabases per day ^5^.

Numerous methods have been developed for the efficient analysis of the increasingly large nanopore datasets. However, current methods to reduce the data storage footprint are extremely limited. Nanopore runs uploaded to online repositories, such as the European Nucleotide Archive, are bundled into a tarball, a process which facilitates upload as a single file, but does not decrease file size. Moreover, ONT runs bundled into a tarball (which could then be compressed using traditional means) are not able to be read by any existing nanopore analysis tools. Moreover, traditional compression technologies are poorly adapted to the needs of the individual user, many of whom have no need for a large portion of the data stored by ONT’s basecallers. Therefore, we developed Picopore, a tool for reducing the storage footprint of the ONT runs without preventing users from using their preferred analysis tools. Picopore uses a combination of storage reduction techniques, including built-in dynamic compression in the HDF5 file format, reduction of data duplication, efficient allocation of memory within the file, and the removal of intermediate data generated during basecalling, which is deemed unnecessary by the end-user.

## Methods

### Implementation

Picopore is developed using the Python h5py module ( http://www.h5py.org/), an interface to the HDF5 file format ( http://www.hdfgroup.org/HDF5), used by ONT under the FAST5 file extension. Picopore implements a number of different compression methods, a selection of which are applied according to user preferences, before using HDF5’s h5repack to rebuild the file according to the reduced file size requirements.

### Compression techniques

***Built-in GZIP compression.*** The HDF5 file format allows for both files and datasets within files to be written using a number of different compression filters, the most universally implemented being GZIP. GZIP applies traditional compression to the data stored in the HDF5 file with choices of compression level between 1 and 9. ONT’s default compression uses GZIP at level 1; Picopore increases this to level 9 in order to decrease disk space usage.

***Dynamic memory allocation for variables.*** Data stored in the HDF5 file format uses fixed-size file formats provided by NumPy, which provides a vast array of options for storing integers, decimals and strings within high-dimensional datasets ^6^. ONT’s native data is written using the largest data types provided by NumPy: 64-bit integers, 64-bit decimals, and "variable-size" strings. Picopore vastly reduces disk space usage by analyzing each dataset to determine the minimum number of bytes required by a given variable in the file, changing the data type accordingly.

***Collapsing of file structure.*** The advantage of the HDF5 file format is that it provides a file directory-like storage format for datasets and properties, making reading and writing to the files straightforward and easy to understand. However, the inherent nature of the highly-structured file format requires HDF5 to allocate slots of memory to "groups", which represent the internal directory structure of the file. Picopore reduces the disk space used by this file metadata by collapsing the directory structure, while retaining the option for users to reverse this action when tools that only recognize the original file format are required.

***Indexing of duplicated data.*** ONT’s most widely used basecalling software, the cloud-based Metrichor service ( https://metrichor.com/s/), performs feature recognition (or "event detection"). This segments the electrical signal representing each nanopore read into events, each of which represents a period of time when the DNA was stationary in the nanopore. These events are then converted into basecalled data, which provides a single k-mer (at present a 5-mer) of DNA representing the bases in the nanopore contributing to the signal at that time. Each event corresponds to a single row in the basecalled dataset, and both the event detection and basecalled datasets thus store the mean signal, standard deviation, start time and length of the event. Picopore reduces disk space usage by indexing the basecalled dataset to the event detection dataset, removing the duplicated data while retaining the option for users to reverse this action when tools that require access to this data are required.

***Removal of intermediate data.*** The primary function of all basecalling software is to generate a FASTQ file containing the genomic sequence and associated quality scores representing the read stored in each FAST5 file. While some software tools, such as nanopolish ^7^ and nanoraw ^8^, do make use of the signal, event detection and basecalled datasets, the large majority of analyses, including alignment, assembly and variant calling, simply require access to the FASTQ data. Picopore allows users to remove the intermediate data generated during the process of converting raw signal to FASTQ, while retaining the signal data, should they ever want to re-basecall the run to attain improved FASTQ data or to access this intermediate data at a later stage.

### Operation

***Requirements.*** Picopore is built in Python 2.7 ( www.python.org) and runs on Windows, Mac OS and Linux. It requires the following Python packages:

- h5py 2.2.0 or later
- future
- watchdog 0.8.3 or later

In addition, Picopore requires HDF5 1.8.4 or newer, with development headers ( libhdf5-dev or similar), including the binary utility h5repack, which is included therein.

***Installation.*** The latest stable version of Picopore is available on PyPi and bioconda (see Software availability). It can be installed according to the following commands:

Linux and Mac OS: pip install picopore

Windows: conda install picopore -c bioconda -c conda-forge

Picopore can also be installed from source (see Software availability) using the command python setup.py install.

***Execution.*** Picopore is run from the command-line as a binary executable as follows:

picopore --mode lossless --prefix shrunk [...] /path/to/fast5/

Picopore accepts both folders and FAST5 files as input. If a folder is provided, it will be searched recursively for FAST5 files, and all files found will be considered as input.

There are three modes of compression available, each of which performs a selection of the techniques described above.

- lossless: performs built-in GZIP compression and dynamic memory allocation for variables. This mode is both fast and allows continued analysis of data by any existing software.
- deep-lossless: performs lossless compression, as well as collapsing of file structure and indexing of duplicated data. This mode obtains the best compression results without removing any data, but comes at the cost of requiring reversion before most software tools can analyse the data.
- raw: performs lossless compression, as well as removal of intermediate data, partially reverting files to the "raw" pre-basecalled file format. This mode is fast, obtains the best filesize reduction, and allows continued analysis by tools that extract FASTQ and related data, but comes at the cost of removing intermediate basecalling data required for some niche applications, such as nanopolish, which cannot be retrieved by Picopore (but can be regenerated using basecalling software.)

Optional arguments include:

--revert: reverts lossless compressed files to their original state to allow high-speed access at the cost of disk usage;
--realtime: watches for file creation in the given input folder(s) and performs the selected mode of compression on new files in real time to reduce the footprint of an ongoing MinION run;
--prefix: allows the user to specify a filename prefix to prevent in-place overwriting of files;
--group: allows the user to select only one of the analysis groups on files that have been processed by multiple basecallers;
--threads: allows the user to specify the number of files to be processed in parallel.

## Results

To demonstrate the effectiveness of Picopore’s compression, we ran all three modes of compression on four toy datasets of 40 FAST5 files run using the R9 SQK-RAD001 (R9_1D), R9 SQK-NSK007 (R9_2D), R9.4 SQK-RAD002 (R9.4_1D) and R9.4 SQK-LSK208 (R9.4_2D) protocols. The files for the toy datasets were chosen randomly from the *pass* folder of four MinION datasets generated at the Australian Genome Research Facility. For the R9_1D dataset, DNA was extracted from the lung of a juvenile 129/Sv mouse using the DNeasy Blood and Tissue kit (Qiagen). For the R9_2D, R9.4_1D and R9.4_2D datasets, DNA was extracted from a culture of *escherichia coli* K12 MG1655 using the Blood & Cell Culture DNA Kit (Qiagen). Quality control performed by visualisation on the TapeStation (Agilent). Run metadata is shown in Table 1.

**Table 1.** Metadata for MinION datasets sampled to produce toy datasets.

Name	Chemistry	Protocol	MinION ID	Flowcell ID	Sample	Strain
R9_1D	R9 1D	SQK-RAD001	MN17324	FAD24340	Mouse	129/Sv
R9_2D	R9 2D	SQK-NSK007	MN17324	FAD24193	*Escherichia coli*	K12 MG1655
R9.4_1D	R9 1D Rapid	SQK-RAD002	MN17324	FAF04136	*Escherichia coli*	K12 MG1655
R9.4_2D	R9 2D	SQK-RAD002	MN17324	FAF04232	*Escherichia coli*	K12 MG1655

**Figure 1.**
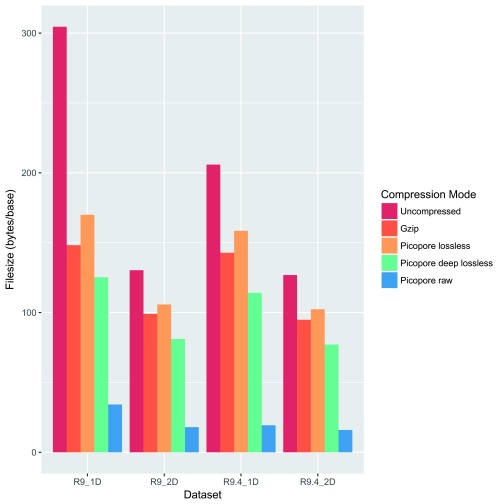
Size of tarball containing FAST5 files compressed using various methods.

**Figure 2.**
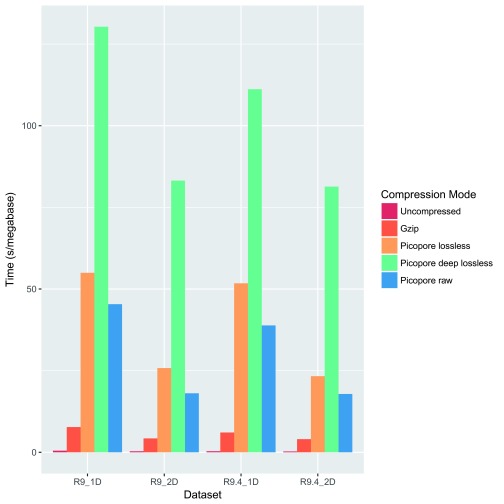
Time taken for single-thread compression and tarring of FAST5 files using various methods.

Each file was compressed and tarred using each of five methods: no compression, gzip (applied after tarring, as per convention), picopore lossless, picopore deep-lossless and picopore raw. Each of these methods was run on a single core, and results were normalised for the number of bases in each dataset, obtained using poretools stats ^9^. Figure 1 shows that lossless achieves only slightly less compression than gzip, giving an average reduction in size of 25% compared to gzip’s 32%, while deep-lossless and raw perform significantly better, giving average reductions in size of 44% and 88%, respectively. A dependent sample t-test was run on individual compressed file sizes. Table 2 shows that each successive method of compression (excluding gzip, which does not compress individual files) gives a significant reduction in size from the previous. Figure 2 shows that while all of Picopore’s compression methods are much slower than gzip, raw is the fastest of these, followed by lossless and deep-lossless. Note that the tarring time makes up a maximum of 0.5 s/megabase in each case and is largely negligible.

**Table 2.** Significance of difference in size of files compressed with different methods using a dependent sample t-test.

Mode 1	Mode 2	t-statistic	p-value
uncompressed	lossless	33.58	< 10 ^−15^
lossless	deep-lossless	20.14	< 10 ^−15^
deep-lossless	raw	17.69	< 10 ^−15^

**Figure 3.**
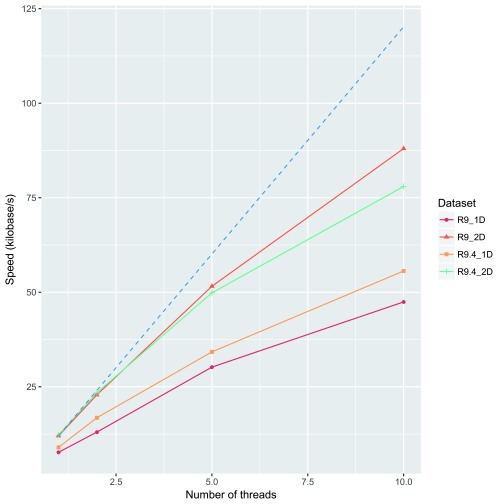
Speed of deep lossless compression of FAST5 files using multiple threads. The dotted blue line shows the theoretical linear maximum increase in speed for the R9 2D run.

To demonstrate the effectiveness of Picopore’s multithreading, we ran deep-lossless, the most computationally expensive of the Picopore compression methods, on each dataset using 1, 2, 5, and 10 cores. Figure 3 shows an almost linear improvement in speed, showing that even on a small dataset, the multithreading overhead is relatively small.

## Discussion

It is clear that, due to the enormous reduction in disk space and low total time requirements, Picopore’s raw compression is the optimal compression mode for users who have no need for the intermediate event detection and basecalling data. The superior running speed of lossless compression over deep lossless compression may mean that this is the preferred method for users who wish to retain all data and need to compress the data in real-time; however, for users with these data retention requirements who wish to store data longterm on their file server, for whom speed of compression is not an issue, deep lossless compression provides the best option. All Picopore compression methods provide significant improvements over uncompressed or traditionally compressed files, and lossless and raw methods carry the added benefit that files can be processed using analysis tools in compressed form.

Although the compression has a high CPU cost, the capability of Picopore to run on multiple threads signifies that, if computing resources are available, the files can be compressed in a reasonably short period of time. Finally, extrapolating the running time per file to a real-time run over 48 hours, Picopore has the capability to run lossless (<= 55 s/Mb) and raw (<= 45 s/Mb) modes on a single core in real time for flowcell yield up to 3Gb (57s/Mb). While deep-lossless requires multiple cores to keep pace with the MinION data generation, this adds little to the overall computational cost, and can reach real-time speed with just five cores (<= 33 s/Mb). Note that experienced users of the MinION have reported single flowcell yields of above 12 Gb ( http://omicsomics.blogspot.com.au/2017/03/catching-up-on-oxford-nanopore-news.html), for which lossless and raw compression would also require multithreading. As data generation continues to increase in scale, further gains could be made by incorporating the compression methods used in Picopore into the basecalling software itself.

As of the 17th of March 2017, ONT announced that the version 1.5 of their MinKNOW software will not store the intermediate data by default ( https://nanoporetech.com/sites/default/files/s3/MinION-Computer-Requirements-March-17_Final.pdf), effectively mimicking Picopore’s raw compression mode. Picopore’s three modes of compression will be maintained for use on datasets generated before the upcoming release of MinNOW 1.5, and for those users who choose to store the event data beyond this point.

## Conclusions

ONT’s MinION and PromethION sequencing devices promise to produce increasingly large datasets as the technology progresses toward commercial release. The disk space required to run and store one or multiple datasets from these poses a problem for service providers and users alike; Picopore provides three different solutions that cater to the different needs of users.

Although the trade-off between data retention, computing time and disk space is a compromise that cannot be perfectly resolved, Picopore provides user options to reduce their ONT datasets to the minimum viable size based on their intended use. This may involve real-time compression for laptop disk space concerns, reduction of bandwidth usage for transfer of datasets between laboratories, or reduction of the storage footprint on shared data servers.

## Data availability

The data referenced by this article are under copyright with the following copyright statement: Copyright: © 2017 Gigante S

Data associated with the article are available under the terms of the Creative Commons Zero "No rights reserved" data waiver (CC0 1.0 Public domain dedication). 

The toy dataset used for the analyses in this paper is available on Zenodo: Toy datasets for compression by Picopore, doi: 10.5281/zenodo.321957.

## Software availability

Software for Linux or Mac OS available from: https://pypi.python.org/pypi/picopore

Software for Linux, Mac OS and Windows available from: https://anaconda.org/bioconda/picopore

Source code available from: https://github.com/scottgigante/picopore

Archived source code from: https://dx.doi.org/10.5281/zenodo.438509

License: GPLv3

## Author endorsement

Chris Woodruff confirms that the author has an appropriate level of expertise to conduct this research, and confirms that the submission is of an acceptable scientific standard. Chris Woodruff declares he has no competing interests. Affiliation: Visiting Scientist at Bioinformatics Division of Walter and Eliza Hall Institute of Medical Research, Parkville, Victoria, Australia
