# Supplementary material for: Picopore: A tool for reducing the storage size of Oxford Nanopore Technologies datasets without loss of functionality
Source: F1000Res. 2017 Sep 28;6:227. Originally published 2017 Mar 7. [Version 3] doi: 10.12688/f1000research.11022.3 (PMC5365225; doi:10.12688/f1000research.11022.3)
Supplement: Peer review history

# Peer review: Referee response for version 3

**DOI:** 10.5256/f1000research.13857.r26511

**Published:** 2017-09-29

**Authors:** David A. Eccles

**Affiliations:** 1 Malaghan Institute of Medical Research, Wellington, New Zealand; 2 Gringene Bioinformatics, Wellington, New Zealand

No further comments.

I have read this submission. I believe that I have an appropriate level of expertise to confirm that it is of an acceptable scientific standard.

# Peer review: Referee response for version 2

**DOI:** 10.5256/f1000research.12305.r21836

**Published:** 2017-04-13

**Authors:** David A. Eccles

**Affiliations:** 1 Malaghan Institute of Medical Research, Wellington, New Zealand; 2 Gringene Bioinformatics, Wellington, New Zealand

The updated version of Picopore fixes all the issues that I reported previously.

I have read this submission. I believe that I have an appropriate level of expertise to confirm that it is of an acceptable scientific standard.

# Peer review: Referee response for version 1

**DOI:** 10.5256/f1000research.11886.r20757

**Published:** 2017-03-21

**Authors:** David A. Eccles

**Affiliations:** 1 Malaghan Institute of Medical Research, Wellington, New Zealand; 2 Gringene Bioinformatics, Wellington, New Zealand

It's quite difficult to knock out a useful software tool for Oxford Nanopore devices before they change the protocol, or release their own tool that does similar things. Scott Gigante has done an admirable job in this regard by developing and publishing a much-needed tool within the space of time between two updates of the MinKNOW software (v1.4 -> v1.5). While the newest version of MinKNOW no longer produces event data by default in the FAST5 files (substantially reducing file sizes), Scott's tool will still be useful for existing file sets and event-called files in the future.

**Manuscript**

 The manuscript is sufficiently verbose for a short software release publication: explaining modes of operation, demonstrating differences in file compression in different modes, and showing processing speed on different datasets.

**Title / abstract**

 The title and abstract sufficiently summarise the manuscript

**Introduction**

- "ONT'" -> "ONT's"
- "The drastic increase... the limiting factor in uptake of the technology" -- cite, alter, or remove. My experience is that storage space is only an issue for *existing* users of the technology, not people deciding whether or not to use the MinION.

**Operation**

- At least one running example in the 'Execution' section would be useful, similar to the Usage section of the pypi repository. Something like this:
  'picopore --mode lossless --prefix shrunk '

**Discussion**

- The introduction suggests ONT's internal runs are approaching 2M reads, yet the discussion suggests 0.5M reads per run. This is the difference between real-time processing and almost a week of waiting for processing to finish.
- The discussion mentions future potential capabilities for basecalling software, but not Picopore itself. Are there any planned updates on the horizon?

**Software testing**

 The current version of Picopore (installed by *pip* on 2017-Mar-17) had a few minor issues on my system that would prevent most users from being able to run the software. Once these issues were dealt with, Picopore was able to substantially reduce the file size of two FAST5 files with wildly different internal structures, while retaining important base call and raw signal information.

**Installation**

 The program appeared to install fine on my Debian Linux desktop by running ' *pip install picopore*', as per the manuscript instructions. Unfortunately there was a problem with module import when showing the help dialog:

     $ picopore -h

     Traceback (most recent call last):

       File "/usr/local/bin/picopore", line 7, in

         from picopore.__main__ import main

       File "/usr/local/lib/python2.7/dist-packages/picopore/__main__.py", line 22, in

         from picopore.parse_args import parseArgs, checkSure

       File "/usr/local/lib/python2.7/dist-packages/picopore/parse_args.py", line 20, in

         from builtins import input

     ImportError: No module named builtins

 Commenting out the offending line in 'parse_args.py' fixed this error.

**Use of other data**

 Instead of using the provided data, I did a stress test of sorts on Picopore by running it on two files which were put in subdirectories of a parent directory:

1. A 2kb R7.3 tomato read produced by me in March 2016 (channel 342, read 13) [ David Eccles' read]
2. A 771kb R9.4 E. coli read produced by Nick Loman and Josh Quick in March 2017 [ Nick Loman & Josh Quick's read]

**Equivalence testing**

 It is appreciated that Picopore includes a test for equivalence to make sure information is retained. Picopore was able to recursively descend through the directories, but the deep-lossless equivalence test reported failure for both of these sequences. In the case of the second file, it appears that the only failure was a missing //Picopore directory (which should probably be excluded from the failure modes):

**- Equivalence test 1**

     $ picopore --prefix pico_ -t 10 --test --mode deep-lossless tested_picopore

     Performing deep lossless compression on 2 files...

     No conversion path for dtype: dtype('     Complete.

     Original size:   67048849

     Compressed size: 66616200

     Checking equivalence of /home/gringer/bioinf/reviews/tested_picopore/1/lambda_TEDxWellington_DavidEccles_3637_1_ch342_read13_strand.fast5 (file 1) and /home/gringer/bioinf/reviews/tested_picopore/1/picopore.test.lambda_TEDxWellington_DavidEccles_3637_1_ch342_read13_strand.fast5 (file 2)...

     Failure: //Analyses missing from file 2

     Failure: //Raw missing from file 2

     Failure: //Sequences missing from file 2

     Failure: //UniqueGlobalKey missing from file 2

     Failure: //Picopore missing from file 1

     Traceback (most recent call last):

       File "/usr/local/bin/picopore", line 11, in

         sys.exit(main())

       File "/usr/local/lib/python2.7/dist-packages/picopore/__main__.py", line 80, in main

         runTest(args)

       File "/usr/local/lib/python2.7/dist-packages/picopore/__main__.py", line 63, in runTest

         checkEquivalent(f, compressedFile)

       File "/usr/local/lib/python2.7/dist-packages/picopore/test.py", line 72, in checkEquivalent

         recursiveCheckEquivalent(file1, file2, group.name)

       File "/usr/local/lib/python2.7/dist-packages/picopore/test.py", line 56, in recursiveCheckEquivalent

         recursiveCheckEquivalent(file1, file2, "/".join([name, key]))

       File "/usr/local/lib/python2.7/dist-packages/picopore/test.py", line 50, in recursiveCheckEquivalent

         if not attr2[key] == value:

     ValueError: The truth value of an array with more than one element is ambiguous. Use a.any() or a.all()

**- Equivalence test 2**

     $ picopore --prefix pico_ -t 10 --test --mode deep-lossless tested_picopore/2

     Performing deep lossless compression on 1 files...

     No conversion path for dtype: dtype('     Complete.

     Original size:   65686831

     Compressed size: 65688039

     Checking equivalence of /home/gringer/bioinf/reviews/tested_picopore/2/loman_771kb_ch181_read4882_strand.fast5 (file 1) and /home/gringer/bioinf/reviews/tested_picopore/2/picopore.test.loman_771kb_ch181_read4882_strand.fast5 (file 2)...

     Failure: //Picopore missing from file 1

     Complete.

**Confirmation dialog**

 The confirmation of writing files is also a great idea, but produces an error when both responses are given. I wonder if this is due to python version incompatibilities (and my attempted prior bugfix):

**- Confirmation test 1**

     $ picopore --prefix pico_ -t 10 --mode raw tested_picopore

     Performing raw compression with FASTQ and no summary on 2 files...

     Are you sure? (yes|no): no

     Traceback (most recent call last):

       File "/usr/local/bin/picopore", line 11, in

         sys.exit(main())

       File "/usr/local/lib/python2.7/dist-packages/picopore/__main__.py", line 84, in main

         run(args.revert, args.mode, args.input, args.y, args.threads, args.group, args.prefix, args.fastq, args.summary)

       File "/usr/local/lib/python2.7/dist-packages/picopore/__main__.py", line 34, in run

         if y or checkSure():

       File "/usr/local/lib/python2.7/dist-packages/picopore/parse_args.py", line 109, in checkSure

         response = input("Are you sure? (yes|no): ")

       File "", line 1, in

     NameError: name 'no' is not defined

**- Confirmation test 2**

     $ picopore --prefix pico_ -t 10 --mode raw tested_picopore

     Performing raw compression with FASTQ and no summary on 2 files...

     Are you sure? (yes|no): yes

     Traceback (most recent call last):

       File "/usr/local/bin/picopore", line 11, in

         sys.exit(main())

       File "/usr/local/lib/python2.7/dist-packages/picopore/__main__.py", line 84, in main

         run(args.revert, args.mode, args.input, args.y, args.threads, args.group, args.prefix, args.fastq, args.summary)

       File "/usr/local/lib/python2.7/dist-packages/picopore/__main__.py", line 34, in run

         if y or checkSure():

       File "/usr/local/lib/python2.7/dist-packages/picopore/parse_args.py", line 109, in checkSure

         response = input("Are you sure? (yes|no): ")

       File "", line 1, in

     NameError: name 'yes' is not defined

 The 'input' function in my version of python does an evaluation after reading input; replacing 'input(...)' with 'raw_input(...)' fixed this error.

 After these errors were fixed enough to allow the code to proceed, Picopore was able to successfully strip out event data (in 'raw' mode) from both the R7.3 and R9.4 FAST5 files, while retaining called FASTQ sequences and raw signal (i.e. it did what it said on the box). File sizes were reduced from 1.3MB down to 577kB for the R7.3 file, and 63Mb down to 9.8MB for the R9.4 file.

 Picopore also retained the 'Model' section from the R7.3 FAST5 files, indicating that it probably does a blacklist removal of known analysis components and retains unknown things in the file hierarchy; this should ensure that Picopore will be reasonably forward-compatible for future file format changes even without updates.

**Threaded mode**

 I tried to run Picopore on the toy dataset in single-threaded mode, broke out of it because it was taking too long, then restarted in threaded mode and realised I wanted to stop *that* as well [Picopore leaves temporary files of a predictable name in the working directories that are not deleted on failure, and I had not deleted them between runs]. Unfortunately, when running Picopore in threaded mode, I was not able to break out of the running program (and needed to kill it using another console).

**Equivalence check on included dataset**

 Running the equivalence check in *lossless* mode on the provided toy dataset produced no errors. No additional testing was done on the toy dataset.

I have read this submission. I believe that I have an appropriate level of expertise to confirm that it is of an acceptable scientific standard.

# Response

**Published:** 2017-04-10

**Authors:** Scott Gigante

**Affiliations:** Walter & Eliza Hall Institute of Medical Research, Australia

Dear Dr. Eccles,

 Thank you for your review.

 I have amended the manuscript according to your suggested revisions.

 You note that an equivalent to Picopore's *raw* compression is now the default behaviour for MinKNOW v1.5. I will continue to examine the output from the latest version of MinKNOW to find new mechanisms for size reduction; however, in the short term, I anticipate that Picopore's major use cases will be to reduce the size of older datasets, and to reduce the size of datasets produced by power users who continue to use MinKNOW with event data enabled.

 Thank you also for your extensive testing of Picopore. The errors you pointed out have been resolved in the latest version of Picopore, available on Pypi, Bioconda and Github.

 Thank you once again for your comments.

 Kind regards,

 Scott Gigante

# Peer review: Referee response for version 1

**DOI:** 10.5256/f1000research.11886.r20761

**Published:** 2017-03-20

**Authors:** Matthew William Loose

**Affiliations:** 1 School of Life Sciences, University of Nottingham, Nottingham, UK

Picopore is a well written package that installs quickly and easily, has clear guidance on its use and addresses a relevant issue in Nanopore sequencing at this time.

 The tools function as described (certainly on OSX).

 I have some reservations about reporting speed in terms of reads/s. I would like to see metrics which take in to account the number of bases being processed per unit time as I suspect the alternative compression options will perform differently by this metric. 

 I also have some reservations about the use of some modes of picopore compression. Users will need to think carefully about the application of modes which are not immediately compatible with existing tool chains. Given recent announcements from Nanopore with respect to provision of off line base calling I suspect a better long term storage of data will be as simple raw files with an associated fastq.gz.

I have read this submission. I believe that I have an appropriate level of expertise to confirm that it is of an acceptable scientific standard.

# Response

**Published:** 2017-04-10

**Authors:** Scott Gigante

**Affiliations:** Walter & Eliza Hall Institute of Medical Research, Australia

Dear Dr. Loose,

 Thank you for your review.

 I would like to note that since the compression modes were tested all on the same dataset of a total of 160 files across four runs, the number of bases is fixed, with a total of 1.7 Mb in each instance. Thus, the comparison of speeds and sizes between compression modes will be equivalent by either metric. However, I agree that bases/s is a more useful metric than reads/s in comparing results between different protocols, and have included this in my analyses.

 In regards to your recommended mode of long term storage, this is indeed my expectation and is Picopore’s recommended mode for end-users. Picopore’s *raw* mode stores by default only the raw signal and the FASTQ, which is compressed using HDF5’s built-in GZIP compression. Picopore’s *lossless* and *deep-lossless* modes are aimed at developers who wish to retain the event data for active use or long-term storage respectively; I acknowledge that these use cases are only suitable for a particular subgroup of users.

 Thank you once again for your comments.

 Kind regards,

 Scott Gigante
